# Insomnia and cardiovascular outcomes

**DOI:** 10.5935/1984-0063.20200109

**Published:** 2021

**Authors:** Monica L. Andersen, Dalva Poyares, Sergio Tufik

**Affiliations:** Departamento de Psicobiologia, Universidade Federal de São Paulo - São Paulo - SP - Brazil.

Insomnia and cardiovascular diseases (CVD) are 2 disorders that have been intensely discussed in the literature due to their prevalence in the world. Chronic insomnia affects 7 to 15% of the adult population^1,2^, while CVD have been the number one cause of death globally for several years^3^. Many factors contribute to the high prevalence of both disorders, including the classical cardiovascular risk factors^4^, and the predisposition of some individuals to develop insomnia^2^. However, changes in modern society, such as increasing work pressure and the introduction of new technologies have resulted in an increase in stress and sleep deprivation. These both factors can have a negative influence on insomnia and cardiovascular health.

Vgontzas et al. (2009)^5^ first reported a link between short sleep duration and CVD and metabolic consequences among individuals with insomnia. In fact, the impact of sleep deprivation on CVD has been well demonstrated^6^. Chronic severe insomnia is associated with hyperarousal and short sleep duration, potentially leading to: 1) hypercortisolemia and increased adrenocorticotropic hormone (ACTH)^5^; 2) increased sympathetic nervous activity, as shown by high levels of norepinephrine and variability in the heart rate of patients with insomnia^7^; and 3) vascular endothelial dysfunction, as indicated by lower flow-mediated dilatation in patients with insomnia^8^. These alterations directly affect cardiovascular function, enhancing the long-term risk of hypertension, coronary disease, and heart failure^7^. Together, short sleep duration (<5h) and insomnia increase the risk of hypertension^5^. Additionally, patients with insomnia have a 45% higher risk of developing CVD. Results from a meta-analysis study have confirmed the importance of insomnia in relation to cardiovascular disease and mortality^9^.

Another important link between these 2 conditions, as described in the review article by Nobre et al. (2021)^10^ is the association between insomnia phenotypes, circadian misalignment, and cardiovascular risk. The authors hypothesized that a putative autonomic nervous system imbalance could play a role in this association. This is a plausible hypothesis, as some patients with severe insomnia associated with hyperarousal have been shown to display a nocturnal non-dipping blood pressure profile^11,12^. Other studies have reported high sympathetic activity among patients with chronic insomnia^7^.

In addition to hyperarousal and sleep deprivation, insomnia is related with high levels of anxiety symptoms and stress, which contribute to the incidence of mental and affective disorders^1,13^. One of the most studied mental disorders - depression - has been extensively investigated in cardiovascular research because, as stated by American Heart Association, this disorder worsens the prognosis of patients with CVD^14^. Although the mechanisms linking depression and CVD are not well elucidated, it seems that endothelial dysfunction may occur in patients with depressive symptoms^15^.

Finally, Nobre et al. (2021)^10^ raised a very interesting issue in their review of the potential role of circadian misalignment in the pathophysiology of cardiovascular consequences among some insomnia patients. Of note, men with insomnia and difficulty initiating sleep have a higher risk of cardiovascular mortality^16,17^. Insomnia symptoms affect daily routine, increase anxiety and stress, predicting mental disorders, and CVD.

CVD have been a serious public health issue, and it is important to recognize insomnia as one of the main factors indicating a poor prognosis in patients with CVD, and ensure its treatment in order to prevent both insomnia and CVD.
